# Electrospun Polycaprolactone (PCL)-Amnion Nanofibrous Membrane Promotes Nerve Regeneration and Prevents Fibrosis in a Rat Sciatic Nerve Transection Model

**DOI:** 10.3389/fsurg.2022.842540

**Published:** 2022-03-18

**Authors:** Jiangbo Bai, Chunjie Liu, Lingde Kong, Siyu Tian, Kunlun Yu, Dehu Tian

**Affiliations:** ^1^Department of Hand Surgery, The Third Hospital of Hebei Medical University, Shijiazhuang, China; ^2^Department of Orthopedics, Tangshan Workers Hospital, Tangshan, China; ^3^Department of Orthopedics, The Third Hospital of Hebei Medical University, Shijiazhuang, China

**Keywords:** nerve repair, amniotic membrane, poly-e-caprolactone, nerve regeneration, fibrosis, macrophage polarization

## Abstract

Functional recovery after peripheral nerve injury repair is typically unsatisfactory. An anastomotically poor microenvironment and scarring at the repair site are important factors impeding nerve regeneration. In this study, an electrospun poly-e-caprolactone (PCL)-amnion nanofibrous membrane comprising an amnion membrane and nonwoven electrospun PCL was used to wrap the sciatic nerve repair site in the rat model of a sciatic nerve transection. The effect of the PCL-amnion nanofibrous membrane on improving nerve regeneration and preventing scarring at the repair site was evaluated by expression of the inflammatory cytokine, sciatic functional index (SFI), electrophysiology, and histological analyses. Four weeks after repair, the degree of nerve adhesion, collagen deposition, and intraneural macrophage invasion of the PCL-amnion nanofibrous membrane group were significantly decreased compared with those of the Control group. Moreover, the PCL-amnion nanofibrous membrane decreased the expression of pro-inflammatory cytokines such as interleukin(IL)-6, Tumor Necrosis Factor(TNF)-a and the number of pro-inflammatory M1 macrophages, and increased the expression of anti-inflammatory cytokine such as IL-10, IL-13 and anti-inflammatory M2 macrophages. At 16 weeks, the PCL-amnion nanofibrous membrane improved functional recovery, including promoting nerve Schwann cell proliferation, axon regeneration, and reducing the time of muscle denervation. In summary, the PCL-amnion nanofibrous membrane effectively improved nerve regeneration and prevent fibrosis after nerve repair, which has good clinical application prospect for tissue repair.

## Introduction

Peripheral nerve injury caused by trauma is a very common clinical disease (1) and the main repair method is direct end-to-end repair by suturing the epineurium (2). However, complete recovery of nerve function is rarely achieved. Peripheral nerve injury may result in partial loss of sensory and motor functions, muscle atrophy, and in severe cases, poor limb function and complete paralysis, reducing the patient's quality of life and resulting in serious social and economic consequences (3, 4). Peripheral nerve regeneration is affected by many factors such as perineural and intraneural scarring, surgical suture method, and age (5, 6). Perineural and intraneural scarring at the repair site are important factors affecting axon regeneration, which reduce the growth rate of axons and limits the number of regrowing axons (7, 8). Therefore, inhibiting scarring proliferation at the repair site is an important measure for nerve function recovery (9, 10), which may allow the target muscle to regain innervation as soon as possible and avoid neuromuscular joint degeneration and muscle atrophy.

Drugs, autologous tissue, natural materials, and synthetic materials are currently used to wrap the nerve repair site, which act as physical barriers between the nerve repair site and surrounding tissues, reducing scarring proliferation and limiting intraneural scarring (11–14). All of these measures have had some success. However, drugs have disadvantages of fast biological absorption and short temporary barrier function. Autologous tissue is limited by insufficient donor sites and scope, and secondary injury to the donor (15). Natural or synthetic materials either have a strong inflammatory response, degrade quickly, or lack support strength and other disadvantages (16, 17). Therefore, the desired materials are biodegradable, with similar structure and function as a natural extracellular matrix, to support cell growth, guide tissue regeneration, and control active factor release to allow for neural sliding. These materials create a microenvironment that promotes axon regeneration and limits scarring formation at the nerve repair site.

An amniotic membrane is a thin membranous structure without blood vessels, lymph cells, or nerves, which has low immunogenicity, a variety of cytokines, and a large amount of collagen and elastin (18–20). An amniotic membrane has anti-inflammatory and anti-fibrosis properties (21), and its unique structure makes it an ideal choice for a nerve repair material. However, an amniotic membrane has a fast degradation rate, poor extrusion resistance, and low mechanical strength (22) that does not provide sufficient space for nerve regeneration. There are measures to improve the structure and properties of the amniotic membrane and overcome congenital defects in the amniotic membrane. Polycaprolactone (PCL) is a non-toxic biodegradable polyester with good histocompatibility and a long degradation time (23, 24). PCL has been used in skin reconstruction, bone tissue engineering, medicinal scaffolds, and medicinal membranes to prevent tendon adhesion (25–27). Electrospinning technology can continuously prepare nanofibers with the unique advantages of a biomimetic natural extracellular matrix. Nanofibers can greatly promote axon regeneration and prevent scarring formation (28).

In order to reduce adhesion and promote nerve repair following peripheral nerve injury surgery, we have invented a novel biomaterial named as PCL-amnion nanofibrous membrane. Our previous study demonstrated that electrospun PCL-amnion nanofibrous membranes obviously alleviate tissue adhesion following neural surgery and accelerate nerve regeneration in a rat model of sciatic nerve compression in order to determine whether this new type of biomaterial can exert beneficial effects in chronic nerve injury animal model (29). In the current study, we explore the effect of PCL-amnion nanofibrous membrane on nerve regeneration and scarring formation at the nerve repair for the recovery of nerve function in a rat sciatic nerve transection model in order to determine whether this new type of biomaterial can exert beneficial effects in acute nerve injury animal model.

## Materials and Methods

### Preparation of the Electrospun PCL-Amnion Nanofibrous Membrane

According to the Declaration of Helsinki and approval from the medical ethics committee of the Third Hospital of Hebei Medical University, an amniotic membrane was provided by the Department of Obstetrics and Gynecology from June 1st of 2021 to June 30th of 2021. After written consent was signed, fresh amniotic membrane was harvested from healthy pregnant women that underwent Cesarean section. The fresh amniotic membrane was immediately washed with physiological saline and laid flat on a nitrocellulose membrane punch with the amniotic epithelial cells directed upwards. The amniotic membrane was prefrozen at −50 to 0°C for 1–2 h, and cooled in a condensation chamber with a cold trap temperature reaching−50 to −30°C. After the vacuum pump was operated for 6–8 h, the freeze-dried amniotic membrane was prepared. One gram PCL (average Mn-80,000; Sigma-Aldrich, USA) and 0.5 g gelatin (Porcine skin, Vetec reagent grade, Sigma-Aldrich, USA) were completely dissolved in 10 ml hexafluoroisopropanol (>99.5%, Shanghai Nortel Co., Ltd.) by electro-magnetic stirring, defoaming, and stirring using a magnetic bar for 15 h. The operating temperature was 25°C and the humidity was 60%; the polymer solution was placed in a 2 ml syringe pump with a blunt needle diameter of 0.7 mm. The solution was delivered at flow rate of Q = 1.0 mL/h and a voltage of 13 kV. As the solution was ejected from the needle, charged PCL nanofibers traversed a distance of 15 cm and were deposited on the two surfaces of the freeze-dried amnions. The PCL-amnion nanofibrous membranes were dried overnight in a vacuum (29). The PCL-amnion nanofibrous membranes were sterilized by cobalt 60 irradiation before use (30).

### Animals

Adult male Sprague–Dawley rats weighing 200–250 g were provided by Hebei Iviwo Biological Technology Co., LTD. The study was conducted under all protocols approved by the Animal Research Ethical Committee of the Third Hospital of Hebei Medical University and in line with the guidelines for animal care and use (Approval number: Z2021 - 008 - 1).

The rats were housed in a 12 h light/dark cycle at a temperature of 22.1°C and 65–70% humidity. Food and water were available *ad libitum*. All the rats were acclimated to the environment for 1 week prior to surgical procedures. All surgery was performed under general anesthesia with sodium pentobarbital (3%), and all efforts were made to ameliorate animal suffering.

### Experimental Design

A total of 64 rats were randomly divided into PCL-amnion nanofibrous membrane group (*n* = 32) and a Control group (*n* = 32). Two time points (4 and 16 weeks) were used to assess the nerve regeneration. The inflammation and nerve regeneration were examined at 4 weeks. At 4 weeks, forty rats were used for histology (twenty-four, *n* = 12 per group) and qRT-PCR (sixteen, *n* = 8 per group). At 16 weeks, functional nerve recovery was examined in 24 rats. Rats were used for sciatic function index (SFI) and nerve electrophysiological assessment. The CMAP amplitude and latency of the sciatic nerve were measured with a myoelectricity-evoked potential apparatus (Viking Quest, Nicolet, US). The rats were then used for histology (*n* = 12 per group).

### Surgical Procedure

General anesthesia was achieved by intraperitoneal injection of 30 mg/kg pentobarbital sodium for all surgery. The hair of the left thigh was clipped and the surgical area was scrubbed with 2% iodophor. A 3.0-cm long incision was made in the dorsal skin of the left thigh along the femur. The muscles were split and the sciatic nerve was exposed. The sciatic nerve was transversely cut 15 mm distally from the sciatic notch. In the Control group, the proximal and distal nerve stumps were reconnected using 10–0 Prolene epineurial sutures. In the PCL-amnion nanofibrous membrane group, the repaired site was wrapped with the PCL-amnion nanofibrous membrane after the epineurial sutures. The PCL-amnion nanofibrous membrane overlapped each nerve stump by 5 mm. The diameter of the wraps was larger than that of the sciatic nerve to avoid compressing the nerve. After the operation, rats were kept warm and protected from moisture. The rats were fed separately in a single cage to ensure adequate food and water. The incision was disinfected with iodophor twice a day for three consecutive days. The rats were monitored for plantar ulcers or autophagy.

### Early Measurement of Nerve Regeneration (4 Week Time Point)

At 4 weeks, the rats were euthanized by intraperitoneal injection with sodium pentobarbital (150 mg/kg), and the sciatic nerve was carefully re-exposed and systematically assessed for adhesions scored according to Petersen et al. (31). The inflammation at the repair site was evaluated using histologic, immunofluorescence, and molecular analysis. In addition, the nerve regeneration was assessed using histomorphometric analysis. At 4 weeks, the total RNA from the 15-mm long sciatic nerve centered on the repaired site of eight rats in each group were extracted using TRIzol eagent according to the product description.

#### Macrophage Immunofluorescence Assessment

Immunofluorescence staining was performed on 4 μm nerve sections for quantifying the number of macrophages in the nerve repair site and their specific phenotype (M1/M2). After antigen repair, the sections were incubated with diluted normal goat serum for 30 min. The sections were incubated in a humidified chamber at 4°C for 15 h with CD68 monoclonal antibody (Invitrogen, MA5-13324) at a dilution of 1:100, CD206 (Proteintech, 18704-1-AP) at a dilution of 1:100, and iNOS (Bioworld, BS1186) at a dilution of 1:200. The sections were washed in PBS buffer three times and incubated with either a FITC-labeled sheep anti-rabbit IgG (Beyotime, P0186) diluted 1:200 or a Cy3-labeled sheep anti-mouse IgG (Beyotime, A0521) diluted 1:500 in a humidified chamber for 1 h at room temperature. DAPI was dropped on the sections to stain the nuclei for 5 min. Finally, PBS buffer was used to wash the sections four times to remove excessive DAPI. Fluorescence microscopy (Olympus BX53) was used to calculate the number of positive cells per mm^2^ of tissue area. The average number of positive cells for each nerve was expressed as mean ± SD.

#### Masson Staining

To assess collagen deposition at the nerve repair site, Masson staining was performed on the 10 μm nerve sections. The sections were fully dewaxed and soaked in distilled water for 2 min. After washing with tap water for 3–5 min, the sections were stained with Weigert's iron hematoxylin solution for 5–7 min, then washed with running water for 5 min followed by distilled water. The sections were then stained with Biebrich scarlet acid fuchsin solution for 5 min, followed by distilled water. The sections were then stained with phosphotungstic/phosphomolybdic acid for 10 min and aniline blue for 5 min. After washing with distilled water, the sections were washed with 1% acetic acid for 1 min. The sections were then dehydrated with 95% and absolute alcohols. The sections were taken from the nerve at the repair site. An image analysis system (Image J, Nation Institutes of Health) was used to calculate the area of intraneural collagen staining. The intraneural collagen level for each nerve was expressed as mean ± SD.

#### Quantitative RT-PCR

The total RNA from nerve tissue (15 mm sciatic nerve centered on the repaired site) was extracted using TRIzol reagent (Tiagen Biochemical Technology, Beijing, China) according to the product description. The concentration of RNA was measured using a Nanodrop 2000 spectrophotometer (NanoDrop Products, Wilmington, DE). cDNA was generated from RNA using Takara PrimeScript RT reagent kit (Bao Biological Engineering, Dalian, China) per the manufacturer's instructions. The PCR reaction was carried out in the PCR amplification instrument by pre-denaturation at 95°C for 30 s, followed by 40 cycles of denaturing at 95°C for 5 s, and annealing/extending at 60°C for 34 s. Relative gene expression data were analyzed using 2^−ΔΔCT^. The genes and related specific primers are represented in Table 1.

**Table 1:** Primer sequences.

**Primer name**	**Sequence 5'-3'**	**Product length**
r-IL6-F	AGAGACTTCCAGCCAGTTGC	126bp
r-IL6-R	CCTCCGACTTGTGAAGTGGT	
r-TNF-α-F	AGACCCTCACACTCAGATCATCTTC	193bp
r-TNF-α-R	CTCCGCTTGGTGGTTTGCTA	
r-IL10-F	GCAAAGAGAACGCGTGGAAC	143bp
r-IL10-R	GTTCGGTCGGAATAGGTCGG	
R-IL13-F	CTCTCGCTTGCCTTGGTGGT	165bp
R-IL13-R	CAGCTGTCAGGTCCACGCTC	
r-ACTIN-F	AAGTGCGACGTGGACATCCG	109bp
r-ACTIN-R	GGGCGGTGATCTCCTTCTGC	

#### Assessment of Nerve Histomorphometry

Nerve removed 10 mm distally from the repair site were used to assess the regenerating nerve fibers. The nerve specimens were perfused with 4% paraformaldehyde, then washed with phosphate buffer, fixed in 1% osmium tetroxide, dehydrated with ethyl alcohol, and finally embedded in epon. The nerve was sliced into thin sections (thickness: 4 μm) and stained with toluidine blue. An image analysis system (Image J, Nation Institutes of Health) was used to evaluate the entire nerve cross-section (32). Morphometric measurements of the sciatic nerve included: (1) average axon density (N/mm^2^), (2) average fiber diameter (μm), and (3) average myelin sheath thickness (μm). All values were expressed as mean ± SD.

### Late Measurement of Nerve Regeneration (16 Week Time Point)

Functional nerve recovery was assessed by SFI at 4, 8, 12, and 16 weeks and electrophysiological analyses at 12 and 16 weeks. At 16 weeks, the rats were euthanized by intraperitoneal injection with sodium pentobarbital (150 mg/kg), and the degree of the gastrocnemius muscle atrophy and nerve regeneration was evaluated.

#### Sciatic Functional Index

The gastrocnemius muscle functional recovery was assessed by the SFI at 4, 8, 12, and 16 weeks (33). Prior to operation, rats were trained to walk through a dark 100 × 7 cm closed box. Before walking through the dark box, a paper was placed on the bottom of the box. The rat's rear paws were dipped with ink and measurements of the rear paws print were made when the rats walked on the box. The following formula was used to calculate the SFI:


(1) $$\begin{array}{l} \begin{array}{c} SFI\;=\;\frac{-38.3\times (EPL\;-\;NPL)}{NPL}+\frac{109.5\times (ETS\;-\;NTS)}{NTS} \end{array}\\ \begin{array}{c} \;+\frac{13.3\times (EIT\;-\;NIT)}{NIT}-8.8 \end{array} \end{array}$$

The paw length (PL) is the length between the top of the third toe to the heel. The toe spread (TS) is the length between the first to the fifth digit. The intermediary toe spread (IT) is the length between the second and fourth digit. The PL, TS, and IT were measured for both the non-operated (N; “normal”) and operated (E; “experimental”) sides. A SFI value closer to 0 indicates better recovery and a SFI value of −100 indicates complete damage.

#### Electrophysiology

To follow the sciatic nerve functional recovery, the CMAP amplitude and latency of the sciatic nerve in each group were measured with a myoelectricity-evoked potential apparatus (Viking Quest, Nicolet, US) at 12 and 16 weeks. The stimulating needle electrode was placed on the proximal end of the sciatic nerve. The recording electrode was placed on the abductor hallucis plantar. The CMAP amplitude and latency were recorded with the stimulating mode (stimulus intensity 40 mV, frequency 1 Hz, duration 0.2 ms). The normal CMAP amplitude and latency were obtained from the contralateral limb. All values were expressed as mean ± SD.

#### Muscle Atrophy Assessment

The gastrocnemius muscle was assessed grossly and histologically. After the rats were euthanized, the right (non-operated side) and left (operated side) gastrocnemius muscles were carefully resected. Muscle blood was absorbed and the gastrocnemius muscle weight was recorded. The wet weight recovery ratio was calculated as (gastrocnemius muscle wet weight on the operated side/ gastrocnemius muscle wet weight on the non-operated side) × 100% (34).

Histological analysis was performed for a morphological assessment of gastrocnemius muscle atrophy. The sections taken from the mid-portion of the gastrocnemius muscles were used for morphological assessment. After antigen repair, endogenous peroxidase was blocked by 3% hydrogen peroxide for 20 min. The sections were incubated with normal goat serum for 30 min. The sections incubated in a humidified chamber at 4°C for 15 h with laminin antibody (Bioss, bs-0821R) at a dilution of 1:200. The sections were washed in PBS buffer three times and incubated with a FITC-labeled sheep anti-rabbit IgG (Beyotime, P0186) diluted 1:500. DAPI was dropped on the sections and the nuclei was stained for 5 min. Finally, PBS buffer was used to washed the sections four times to remove excess DAPI. Image J software was used to the average single-muscle fiber cross-sectional area (CSA). Morphometric measurements included: (1) the average single-muscle fiber cross-sectional area (μm ^2^) and (2) the single muscle fiber cross-section area distribution. All values were expressed as mean ± SD.

#### Assessment of Nerve Histomorphometry

Sixteen weeks post-surgery, the nerve tissues at the repair site were fixed in 3% glutaraldehyde. The nerve tissues was fixed in 1% osmium tetraoxide solution and then embedded in epoxy resin. Transverse ultra-thin sections were cut from the samples. Sections were stained with 3% uranium acetate and 0.1% lead citrate. Finally, sections were examined with a transmission electron microscope (TEM). Morphometric measurements of the sciatic nerve included: (1) average axon density (N/mm^2^), (2) average fiber diameter (μm), and (3) average myelin sheath thickness (μm). All values were expressed as mean ± SD.

### Statistics

All values were expressed as mean ± SD and analyzed by SPSS Statistics, version 21.0 software. For multiple group comparisons, one-way ANOVA was used. When significant differences were detected, the SNK-q test was used for pairwise comparisons. The *T*-test was used to compared two groups. *P* < 0.05 was considered significant.

## Results

### PCL-Amnion Nanofibrous Membrane vs. Conventional Epineurial Repair, 4 Weeks

#### Gross Examination of the Repair Site

**Figure 1:**
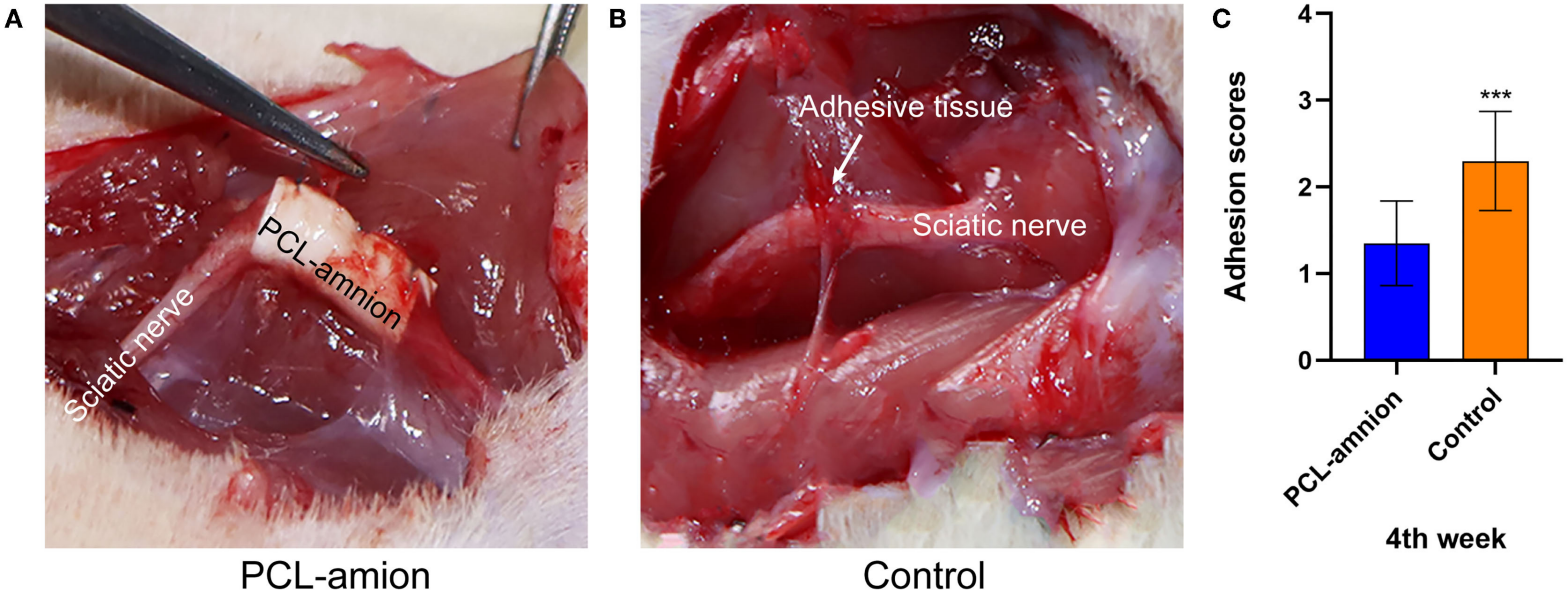
Adhesion assessment of the two groups at four weeks. **(A)** The PCL-amnion nanofibrous membrane group. **(B)** The Control group. **(C)** Adhesion scores of each group at each time point (*P* < 0.05). ***P < 0.001 compared to the PCL-amnion group.

At 4 weeks postoperative, loose adhesions and very little tissue were found around the sciatic nerve in the PCL-amnion nanofibrous membrane group. The surface of the nanofibrous membrane remained smooth (Figure 1A). In contrast, extensive and dense adhesions that required more blunt separation were found around the sciatic nerve in the Control group (Figure 1B). The scores of the PCL-amnion nanofibrous membrane group were less than those of the Control group (*P* < 0.05) (Figure 1C).

#### Inflammatory Reaction at the Repair Site

**Figure 2:**
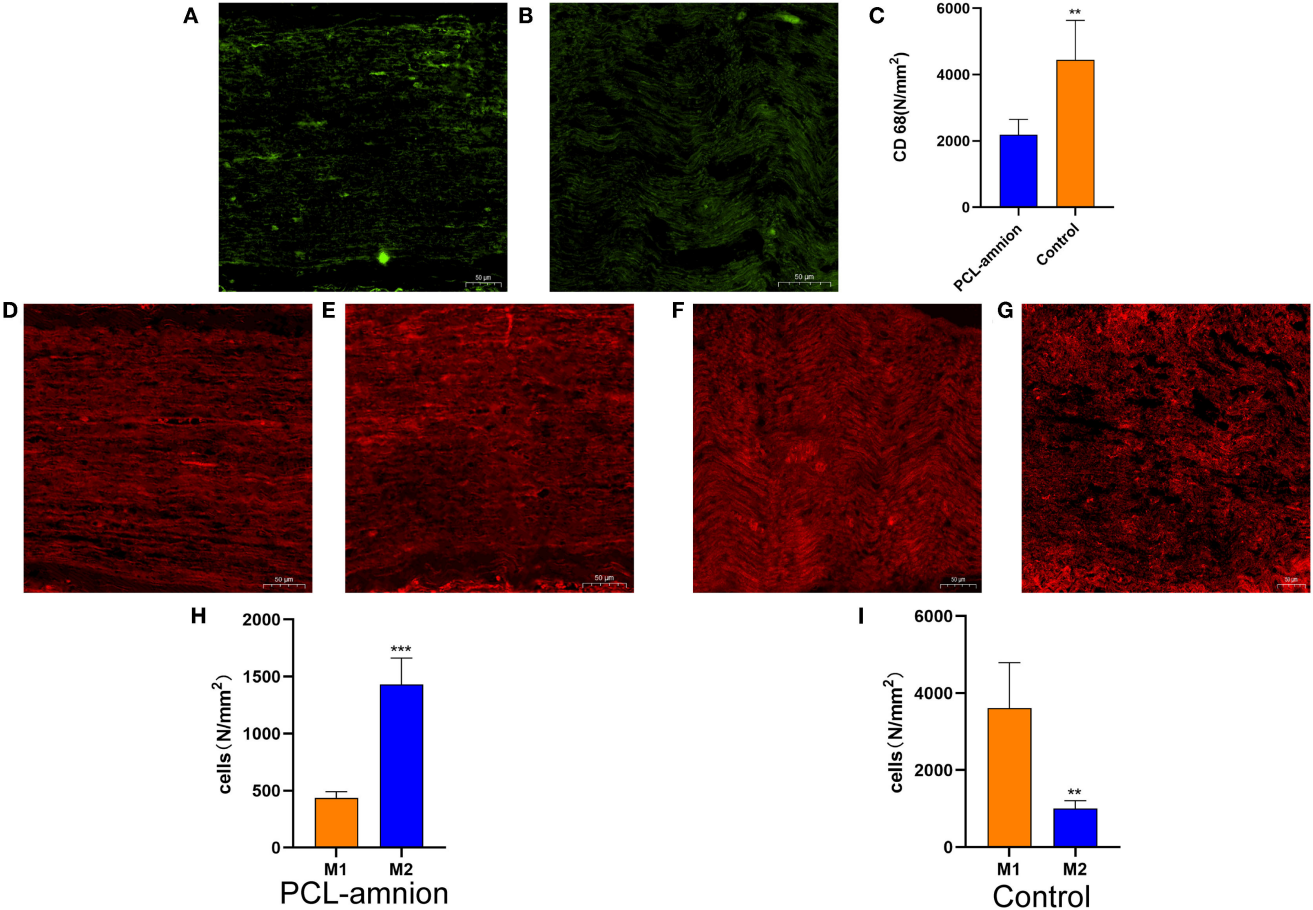
Inflammatory reaction assessment at the repair site. **(A–C)** ED1 macrophages of the sciatic nerve at the repair site. **(A)** The PCL-amnion nanofibrous membrane group. **(B)** The Control group. **(C)** The ED1 macrophage count between the two groups were significantly different (*P* < 0.05). **P < 0.01 compared to the PCL-amnion group. **(D–I)** M1 and M2 of the sciatic nerve at the repair site. **(D)** M1 of the sciatic nerve in the PCL-amnion nanofibrous membrane group. **(E)** M2 of the sciatic nerve in the PCL-amnion nanofibrous membrane group. **(F)** M1 of the sciatic nerve in the Control group. **(G)** M2 of the sciatic nerve in the Control group. **(H)** The M1:M2 ratio in the PCL-amnion nanofibrous membrane group was 1:3 (*P* < 0.05). ***P < 0.001 compared to M1. **(I)** The M1:M2 ratio in the Control group was 3:1 (*P* < 0.05). **P < 0.01 compared to M1.

To evaluate the inflammatory response at the nerve repaired site, ED-1 macrophages were labeled with the anti-CD68 antibody (Figures 2A,B). The number of intraneural macrophages (per mm^2^) in the PCL-amnion nanofibrous membrane group (mean, 2,189 ± 464) was significantly less than in the Control group (mean, 4,441 ± 1,194), (*P* < 0.05) (Figure 2C), indicating that the PCL-amnion nanofibrous membrane inhibited the inflammatory response at the repair site. In addition, macrophages were classified as M1 or M2 by phenotypic characterization. M1 macrophages were labeled with anti-iNOS antibody and M2 macrophages were labeled with anti-CD206 antibody. The number of M1 macrophages (per mm^2^) (mean, 3,611 ± 1,182) was significantly greater than M2 macrophages (per mm^2^) (mean, 1,004 ± 204) in the Control group (*P* < 0.05). However, the number of M2 macrophages (per mm^2^) (mean, 1430 ± 232) was significantly greater than M1 macrophages (per mm^2^) (mean, 437 ± 53) in the PCL-amnion nanofibrous membrane group (*P* < 0.05) (Figures 2D–I).

**Figure 3:**
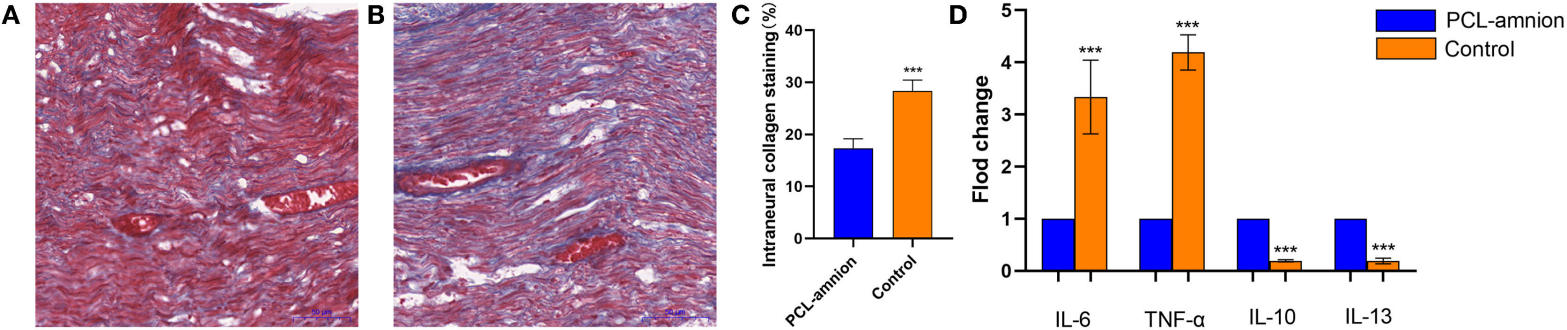
Collagen deposition and quantification of cytokine expression at the repair site. **(A)** The PCL-amnion nanofibrous membrane group. **(B)** The Control group. **(C)** Quantification of collagen at the repair site between the two groups were significantly different (*P* < 0.05). **(D)** The gene expression of proinflammatory cytokines IL6 and TNF-a decreased, and the gene expression of anti-inflammatory cytokines IL10 and IL13 increased in the PCL-amnion nanofibrous membrane group (*P* < 0.05 for each cytokine). ***P < 0.001 compared to the PCL-amnion group.

The amount of collagen at the sciatic nerve repaired site is shown in Figure 3. The mean percentage of intraneural collagen staining (mean, 17.3 ± 1.8%) (Figure 3A) in the PCL-amnion nanofibrous membrane group was significantly less than in the Control group (mean, 28.3 ± 2.1%, Figure 3B) (*P* < 0.05) (Figure 3C).

By analyzing the relative content of cytokine gene expression, the expression of the pro-inflammatory cytokine IL-6 and TNF-α significantly decreased at the repair site in the PCL-amnion nanofibrous membrane group (*P* < 0.05 for each cytokine). Moreover, the expression of the anti-inflammatory cytokines IL-10 and IL-13 significantly increased at the repair site in the PCL-amnion nanofibrous membrane group (*P* < 0.05 for both cytokines) (Figure 3D).

#### Early Evaluation of Nerve Regeneration

**Figure 4:**
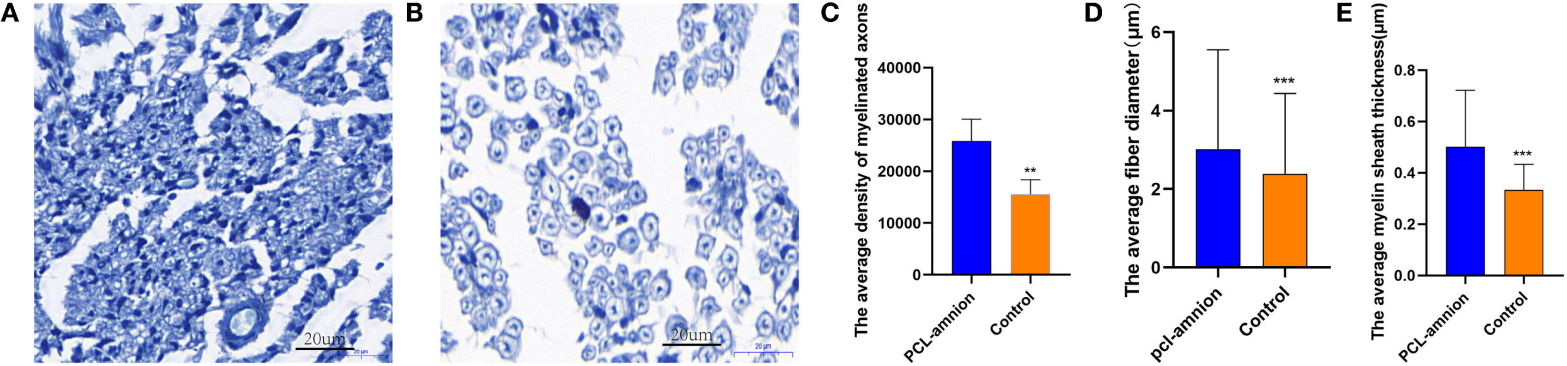
Histomorphometry analysis of nerve regeneration. **(A)** The PCL-amnion nanofibrous membrane group. **(B)** The Control group. **(C–E)** More mature fibers were observed in the PCL-amnion nanofibrous membrane group. **(C)** The average density of myelinated axons was significantly different between two groups (*P* < 0.05). **(D)** The average fiber diameter of myelinated nerve fibers was significantly different between two groups (*P* < 0.05). **(E)** The average myelin sheath thickness of the sciatic nerve was significantly different between two groups (*P* < 0.05). ***P < 0.01 compared to the PCL-amnion group. **P < 0.001 compared to the PCL-amnion group.

In the PCL-amnion nanofibrous membrane group, the average density of myelinated axons was significantly greater than that of the Control group (per mm^2^) (25,864 ± 4,195 vs. 15,555 ± 2,796, *P* < 0.05). Furthermore, the average fiber diameter was significantly greater in the PCL-amnion nanofibrous membrane group in comparison with that in the Control group (3.0 ± 2.5 vs. 2.4 ± 2.1 μm, *P* < 0.05). The average myelin sheath thickness was significantly greater in the PCL-amnion nanofibrous membrane group than that in the control group (0.50 ± 0.22 vs. 0.33 ± 0.10 μm, *P* < 0.05) (Figures 4A–E).

### PCL-Amnion Nanofibrous Membrane vs. Conventional Epineurial Repair, 16 Weeks

#### Sciatic Function Index

**Figure 5:**
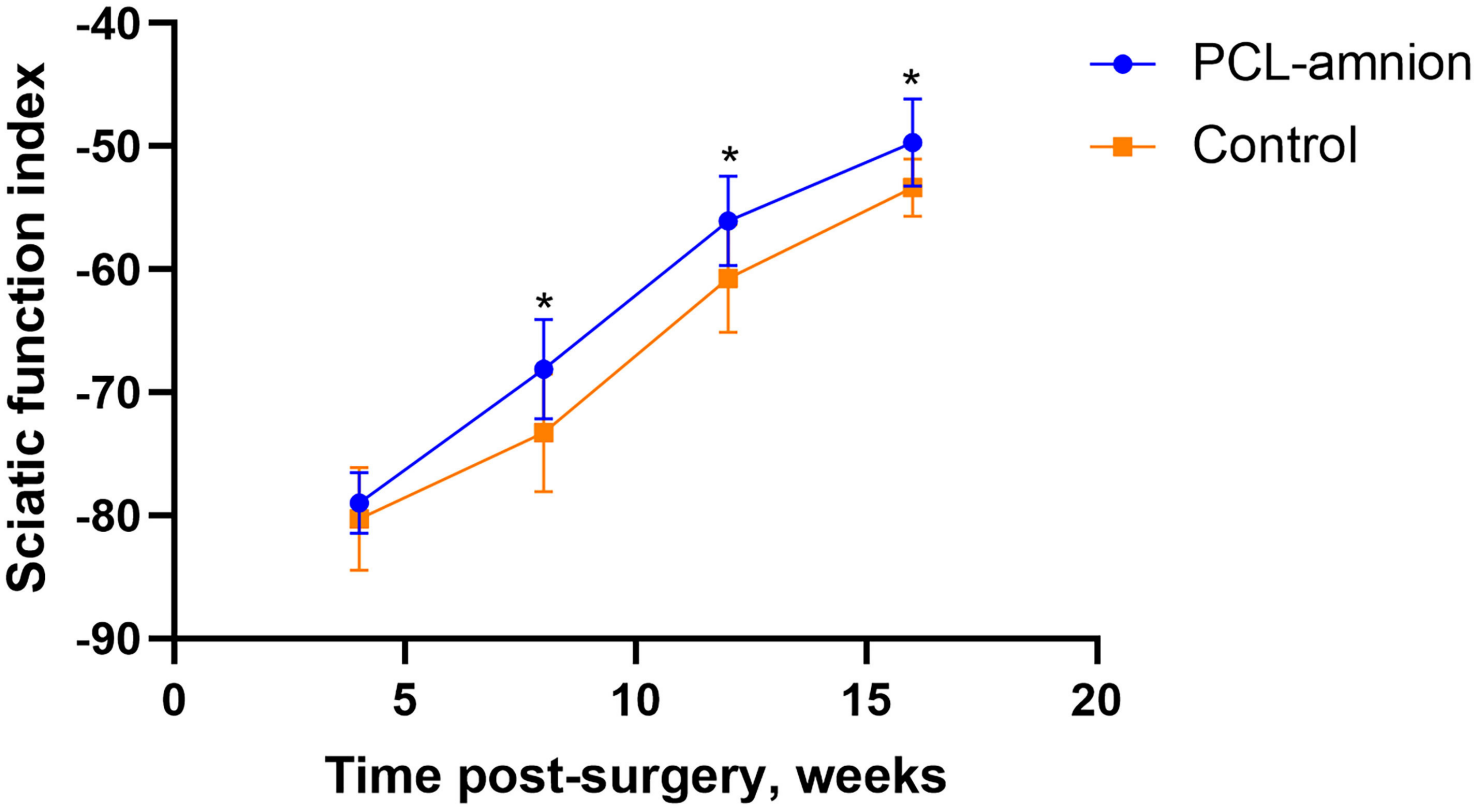
Sciatic function index. The SFI between the two groups was significantly different at 8, 12, and 16 weeks (*P* < 0.05). *P < 0.05 compared to the control group.

The SFI was used to evaluate the function recovery of the gastrocnemius muscle, starting at 4 weeks after never repair. The footprint of the two groups was convenient to calculate SFI. The SFI of each group was similar 4 weeks postoperative (*P* > 0.05). However, at 8, 12, and 16 weeks postoperative, the SFI of the PCL-amnion nanofibrous membrane group was significantly greater than that in the Control group (8 weeks: −68.10 ± 4.07 vs. −73.28 ± 4.78, *P* < 0.05; 12 weeks: −56.05 ± 3.62 vs. −60.74 ± 4.38, *P* < 0.05; 16 weeks: −49.68 ± 3.53 vs. −53.36 ± 2.32, *P* < 0.05) (Figure 5).

#### Electrophysiology

**Figure 6:**
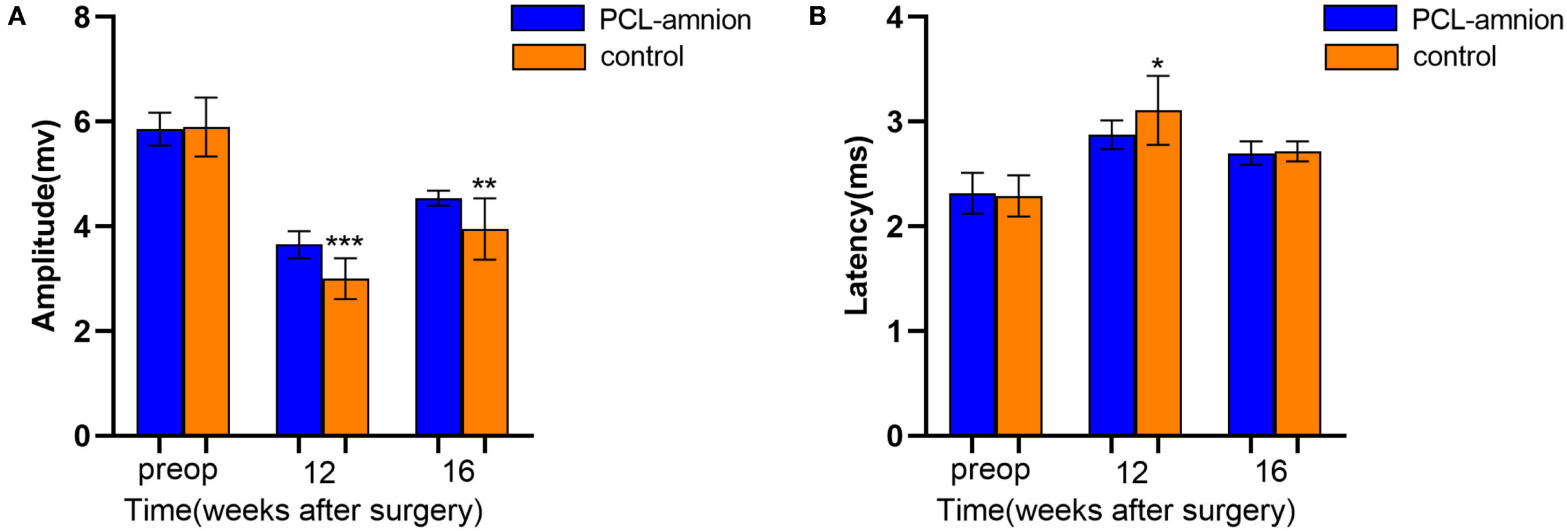
Electrophysiology testing. The motor amplitude and latency were analyzed to assess the functional recovery of the sciatic nerve. **(A)** The PCL-amnion nanofibrous membrane group shows significantly greater amplitude at 12 and 16 weeks (*P* < 0.05). ***P < 0.001 compared to the PCL-amnion group at 12 weeks. ***P < 0.01 compared to the PCL-amnion group at 16 weeks. **(B)** The PCL-amnion nanofibrous membrane group shows significantly greater latency at 12 weeks (*P* < 0.05); however, the latencies between two groups were not significantly different at 16 weeks (*P* > 0.05). *P < 0.05 compared to the PCL-amnion group at 12 weeks.

To follow sciatic functional recovery, the motor amplitude and latency of the sciatic nerve were measured at 12 and 16 weeks after never repair. The motor amplitude in the PCL-amnion nanofibrous membrane group was always significantly greater than that in the Control group (*P* < 0.05) (Figure 6A). At 12 weeks, the motor latency in the PCL-amnion nanofibrous membrane group was significantly less than that in the Control group (*P* < 0.05). Although no significance difference at 16 weeks (*P* > 0.05), the motor latency in the PCL-amnion nanofibrous membrane group was less than that in the Control group (Figure 6B). Therefore, the number of mature nerve fibers re-innervating the target muscle was larger in the PCL-amnion nanofibrous membrane group than in the Control group.

#### Muscle Atrophy

**Figure 7:**
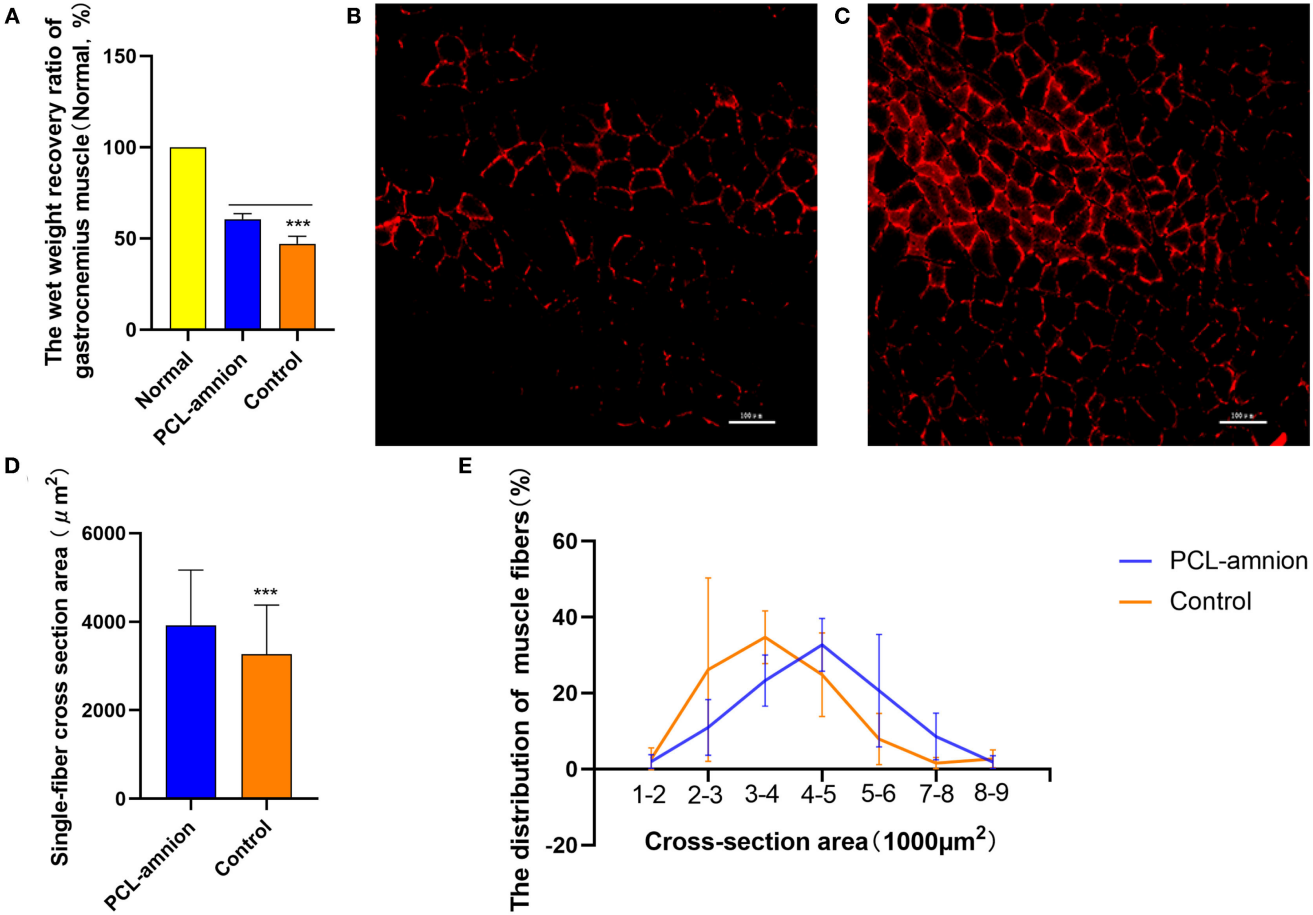
Gastrocnemius muscle analysis. **(A)** Wet weight recovery ratio of gastrocnemius muscle in the PCL-amnion nanofibrous membrane group is significantly higher in comparison with the control group (*P* < 0.05). **(B)** The PCL-amnion nanofibrous membrane group. **(C)** The Control group. **(D)** Average single-fiber CSA. The PCL-amnion nanofibrous membrane group shows significantly greater single-fiber CSA in comparison with the control group (*P* < 0.05). **(E)** Distribution of single-fiber CSA. ***P < 0.001 compared to the PCL-amnion group.

The wet weight recovery ratio of gastrocnemius muscle in the PCL-amnion nanofibrous membrane group was 60.52 ± 3.09% and 47.09 ± 4.05% in the Control group (*P* < 0.05) (Figure 7A).

The anti-laminin antibody was used to delimit the muscle fibers. The average single-fiber CSA was greater in the PCL-amnion nanofibrous membrane group (3,924 ± 1,243 vs. 3,265 ± 1,110 μm^2^, *P* < 0.05) (Figures 7B–D). The frequency distribution of muscle fibers was plotted by single-fiber CSA (Figure 7E). The histogram of the single-fiber area shifted to the right in the PCL-amnion nanofibrous membrane group.

#### Morphometric Measures of Late Nerve Regeneration

**Figure 8:**
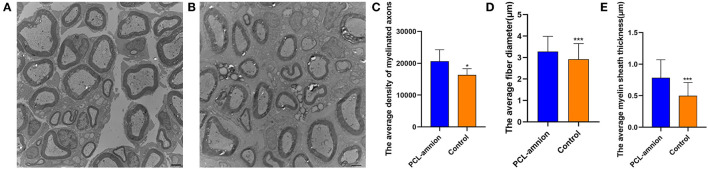
Transmission electron microscopy images of the nerve tissues at 16 weeks. **(A)** The PCL-amnion nanofibrous membrane group. **(B)** The Control group. **(C)** The average density of myelinated axons was significantly different between two groups (*P* < 0.05). **(D)** The average fiber diameter of myelinated nerve fibers was significantly different between two groups (*P* < 0.05). **(E)** The average myelin sheath thickness of the sciatic nerve was significantly different between two groups (*P* < 0.05). *P < 0.05 compared to the PCL-amnion group. ***P < 0.001 compared to the PCL-amnion group.

Sixteen weeks after nerve repair, the average density of myelinated axons was significantly greater than that of the Control group (per mm^2^) (20,627 ± 3,629 vs. 16,334 ± 1,963, *P* < 0.05). Furthermore, the average diameter and myelin sheath thickness of the myelinated nerve fibers were significantly greater in the PCL-amnion nanofibrous membrane group than the Control group (3.3 ± 0.7 vs. 2.9 ± 0.7 μm, *P* < 0.05; and 0.78 ± 0.29 vs. 0.50 ± 0.21 μm, *P* < 0.05) (Figures 8A–E). In the PCL-amnion nanofibrous membrane group, compared to the control group, nerve fibers were more orderly arranged, the myelin lamina was thicker and denser, and the ultrastructure of microtubules and microfilaments was more regular.

## Discussion

The end-to-end epineurial suture remains the standard surgical approach for neurotmesis. In previous studies, researchers mainly studied how to promote nerve regeneration, ignoring various inhibiting factors in nerve repair, such as scarring formation and adhesion at the nerve repair site (35–37). During peripheral nerve injury repair, nerve scarring obstructs the passage of axon regeneration and compresses nerves by adhesion between nerve and surrounding tissue, which reduces the nerve blood supply and hinders nerve regeneration and functional recovery (38, 39). Although rapidly developing micro-surgical techniques promote the recovery of nerve function, some degree of scarring that still exists in the nerve repair site limits the recovery of nerve function (40). Previous studies found that nerve regeneration required an appropriate micro-environment, which promotes nerve regeneration and reduces scarring formation (41).

The amniotic membrane, as a nerve conduit, can promote nerve regeneration and prevent scarring formation (42, 43). The possible mechanism is as follows: the amniotic membrane acts as a barrier function to prevent scarring proliferation and the invasion of inflammatory cells and fibroblasts (22). A closed regeneration chamber can be formed to avoid the loss of nerve factors secreted by nerve injury sites and provide a favorable micro-environment for nerve regeneration, which directionally directs the regenerated axons to distal target organs. An amniotic membrane contains laminin and fibronectin for promoting axon regeneration (44). An amniotic matrix inhibits tissue fibrosis. The amniotic epithelial cells were inactivated and had low immunogenicity by freeze-drying the amniotic membrane. Hua et al. (45) implanted freeze-dried amniotic membranes with amniotic epithelial cells into animals, and found that freeze-dried amniotic membranes showed low immunogenicity which was similar to immune pardoned tissues. An amniotic membrane and Human Amniotic Epithelial Stem Cells (hAESCs) were implanted into an immunotype-mismatched human subcutaneous pocket, and an amniotic membrane and hAESCs survived in the host for a long time without displaying any infiltration or rejection of host immune cells, indicating that amnion-derived cells, including hAESCs, had low immunogenicity (46). However, an amniotic membrane has weak mechanical strength, rapid degradation, and absorption (22). In order to overcome the shortages of amniotic membrane, we have invented a novel type of biomaterial, PCL-amnion nanofibrous membrane, to reduce prevent scarring proliferation and promote nerve regeneration.

Studies have confirmed that macrophages and cytokines play key roles in peripheral nerve regeneration (47–49). At an early stage of nerve repair (1–10 days), M1 macrophages are mainly activated at the nerve injury site, which secretes pro-inflammatory cytokines and molecules inducing tissue damage and condition aggravation (50). However, at the late stage of nerve repair (15–30 days), M2 macrophages are mainly activated, which inhibit the inflammatory response and promote nerve regeneration (51). In this study, we made a new neural scaffold to create a temporary micro-environment for nerve regeneration, and the ordered nano-scaffold was conducive to the activation of macrophages into M2. At 4 weeks, macrophages in the PCL amniotic membrane group were mainly activated to M2 macrophages. In addition, there were changes in the expression of pro-inflammatory and anti-inflammatory cytokines. The expression of the anti-inflammatory cytokines IL-10 and IL-13 at the repair site increased significantly more in the PCL-amnion nanofibrous membrane group than in the Control group. These results indicate that the PCL-amnion nanofibrous membrane regulates the proportion of M1/M2 macrophages, inhibiting inflammatory factors and enhancing the expression of anti-inflammatory cytokine. The Masson's trichrome staining presented in this study showed that the collagen deposition at the repair site was markedly less in the PCL-amnion nanofibrous membrane group than in the Control group. The differential cytokine expression reduced the undesirable immune responses of scarring and fibrosis.

The PCL-amnion nanofibrous membrane has a similar size as matrix collagen fiber in order to facilitate the tissue repair, and the effective scaffold pore structure created by electrostatic spinning technology is to facilitates the transfer of bioactive molecules, nutrients, and metabolic wastes. In this study, the PCL-amnion nanofibrous membrane consists of PCL in the outer layer and amniotic membrane in the inner layer. The amniotic membrane can slowly release TGF-β1, bFGF, PDGF, and NGF cytokines (52). These cytokines continuously diffuses through the pore structure of PCL to the nerve repair site and promotes the proliferation of Schwann cells and axon regeneration. In a previous study, PCL with adipose stem cells was used as a scaffold for nerve regeneration (53). In a rat sciatic nerve transection model, a PCL nanofibrous scaffold loaded by a mesenchymal stem cell condition medium was shown to promote nerve regeneration (54). For repair of sciatic nerve injury, bFGF-chitosan scaffolds were prepared and used to facilitate nerve regeneration (55). At 16 weeks, the axon image showed that there was more mature fibers crossing the suture site in the PCL-amnion nanofibrous membrane group than in the Control group. The nerve fibers were arranged regularly in the PCL-amnion nanofibrous membrane group. Compared with those in the Control group, the axonal diameter and the myelin sheath in the PCL-amnion nanofibrous membrane group were greater and thicker, respectively. In addition, a shorter motor latency time, greater CMAP amplitudes, higher wet weight recovery ratio, and higher single-fiber muscle cross-sectional area were demonstrated in the PCL-amnion nanofibrous membrane group in comparison with control group.

## Conclusions

The present study demonstrated that the PCL-amnion nanofibrous membrane with specific fibers and large pores markedly enhanced Schwann cell proliferation, axonal regeneration, and prevented scar formation at the nerve repair site with the end-to-end epineurial suture. Importantly, the PCL-amnion nanofibrous membrane drove macrophage polarization into the M2 phenotype, which promoted nerve regeneration and functional recovery. Our findings provided a new treatment approach to prevent scar formation at the nerve repair site and promote nerve regeneration, which should aid the design of next generation nerve wraps.

The limitation of the present study is lacking of the tests for immunological safety, cytocompatibility and cytotoxicity of PCL amniotic nanofiber membrane. In the further research work, we conduct the immunological safety, cytocompatibility and cytotoxicity test for our PCL amniotic nanofiber membrane. Moreover, we will continue to explore how macrophages are regulated and activated by the PCL-amnion nanofibrous membrane, how M1 macrophages affected Wallerian degeneration, and M2 macrophages affected Schwann cell proliferation and axonal regeneration. Furthermore, the disadvantage of PCL is its slow rate of degradation. An ideal wrapping material begins degradation after epineurium healing, and nerve fibers cross the repair site. Therefore, PCL and other natural polymer materials can be combined to form a nerve wrap to improve the biodegradation rate.

In summary, our findings provided a new treatment approach to prevent scar formation at the nerve repair site and promote nerve regeneration, which should aid the design of next generation nerve wraps.

## Data Availability Statement

The original contributions presented in the study are included in the article/supplementary materials, further inquiries can be directed to the corresponding author.

## Ethics Statement

The studies involving human participants were reviewed and approved by according to the Declaration of Helsinki and approval from the Medical Ethics Committee of The Third Hospital of Hebei Medical University, an amniotic membrane was provided by the Department of Obstetrics and Gynecology from June 1st of 2021 to June 30th of 2021. The patients/participants provided their written informed consent to participate in this study. The study was conducted under all protocols approved by the Animal Research Ethical Committee of The Third Hospital of Hebei Medical University and in line with the guidelines for animal care and use (Approval number: Z2021 - 008 - 1).

## Author Contributions

DT: conceptualization, funding acquisition, project administration, and writing—review and editing. JB, CL, and LK: data curation. JB, CL, ST, and KY: formal analysis. CL: resources. JB, CL, and KY: software. JB: writing—original draft. All authors contributed to the article and approved the submitted version.

## Funding

This study was supported by the Natural Science Foundation of Hebei Province (H2019206388).

## Conflict of Interest

The authors declare that the research was conducted in the absence of any commercial or financial relationships that could be construed as a potential conflict of interest.

## Publisher's Note

All claims expressed in this article are solely those of the authors and do not necessarily represent those of their affiliated organizations, or those of the publisher, the editors and the reviewers. Any product that may be evaluated in this article, or claim that may be made by its manufacturer, is not guaranteed or endorsed by the publisher.
